# Gut heavy metal and antibiotic resistome of humans living in the high Arctic

**DOI:** 10.3389/fmicb.2024.1493803

**Published:** 2024-10-30

**Authors:** Aviaja Lyberth Hauptmann, Joachim Johansen, Frederik Filip Stæger, Dennis Sandris Nielsen, Gert Mulvad, Kristian Hanghøj, Simon Rasmussen, Torben Hansen, Anders Albrechtsen

**Affiliations:** ^1^SILA Department, Institute of Health and Nature, Ilisimatusarfik – The University of Greenland, Nuuk, Greenland; ^2^Center for Evolutionary Hologenomics, The Globe Institute, The University of Copenhagen, Copenhagen, Denmark; ^3^Novo Nordisk Foundation Center for Protein Research, Faculty of Health and Medical Sciences, The University of Copenhagen, Copenhagen, Denmark; ^4^Department of Health Technology, Technical University of Denmark, Kongens Lyngby, Denmark; ^5^Section for Computational and RNA Biology, Department of Biology, University of Copenhagen, Copenhagen, Denmark; ^6^Department of Food Science, University of Copenhagen, Copenhagen, Denmark; ^7^The Novo Nordisk Foundation Center for Basic Metabolic Research, University of Copenhagen, Copenhagen, Denmark

**Keywords:** Arctic, contaminants, heavy metal resistance, antimicrobial resistance, co-resistance, *mer*-operon

## Abstract

Contaminants, such as heavy metals (HMs), accumulate in the Arctic environment and the food web. The diet of the Indigenous Peoples of North Greenland includes locally sourced foods that are central to their nutritional, cultural, and societal health but these foods also contain high concentrations of heavy metals. While bacteria play an essential role in the metabolism of xenobiotics, there are limited studies on the impact of heavy metals on the human gut microbiome, and it is so far unknown if and how Arctic environmental contaminants impact the gut microbes of humans living in and off the Arctic environment. Using a multiomics approach including amplicon, metagenome, and metatranscriptome sequencing, we identified and assembled a near-complete (NC) genome of a mercury-resistant bacterial strain from the human gut microbiome, which expressed genes known to reduce mercury toxicity. At the overall ecological level studied through α- and β-diversity, there was no significant effect of heavy metals on the gut microbiota. Through the assembly of a high number of NC metagenome-assembled genomes (MAGs) of human gut microbes, we observed an almost complete overlap between heavy metal-resistant strains and antibiotic-resistant strains in which resistance genes were all located on the same genetic elements.

## 1 Introduction

It is well established that the human gut microbiota—the microorganisms residing in the human gastrointestinal tract and their genomic capacity, collectively known as the microbiome—has a tremendous impact on human health and disease. The human gut microbiome plays a multitude of important roles, including the metabolism of otherwise indigestible complex carbohydrates (Gibson et al., 2017), vitamin supplementation (Rowland et al., 2018), and the metabolism of xenobiotics (Koppel et al., 2017). The study of the gut microbiome’s role in mediating toxicity predates high-throughput DNA sequencing methodologies (Rowland, 1988). More recently, it has been demonstrated that the metabolic activity of the human gut microbiota modulates the toxicity of environmental contaminants for the host (Claus et al., 2016; Chiu et al., 2020), and it is recognized that the gut microbiota is a significant, and so far, underestimated factor when evaluating the environmental contaminants’ toxicity (Claus et al., 2016). However, there are still substantial gaps in our knowledge of the gut microbiota’s interactions with heavy metals (HMs) and the resulting toxicological implications (Koppel et al., 2017; Chiu et al., 2020; Duan et al., 2020; Giambò et al., 2021; Ghosh et al., 2023). Studies on the impact of heavy metals on gut microbiota have predominantly focused on animal models (Chiu et al., 2020; Duan et al., 2020), but the number of studies including humans is increasing (Bisanz et al., 2014; Rothenberg et al., 2016, 2019; Caito et al., 2018; Guo et al., 2018; Eggers et al., 2019; Brabec et al., 2020; Shao and Zhu, 2020; Conteville et al., 2023).

Some of the best-studied heavy metal resistance (HMR) genes are those of the *mer-*operon. It is distinguished by being the only bacterial metal resistance system that transforms the toxic target at a large scale (Barkay et al., 2003). In contrast to lead (Pb) and cadmium (Cd) resistance genes, *mer* genes are specific to mercury (Hg). Gut microbial metabolism has the potential to reduce mercury toxicity to the host through the mercuric reductase MerA and the organomercuric lyase MerB, which demethylate Hg (Koppel et al., 2017). Previously, MerA and MerB have been identified in human fecal isolates (Liebert et al., 1997), but only low levels of MerA and no MerB were identified in a human clinical trial with 17 pregnant women (Rothenberg et al., 2016).

HMR genes tend to co-occur with antimicrobial resistance (AMR) genes (Baker-Austin et al., 2006; Vats et al., 2022), a tendency also observed in the human gut (Marshall et al., 1981). In a study comparing different human cohorts, including an Indigenous Wayampi population, there was a significantly higher frequency of Hg-resistant *Escherichia coli* in the gut microbiota of populations exposed to higher levels of Hg, and this group had the highest carriage rate of AMR *E. coli* despite lower exposure to antibiotics suggesting possible co-selection of AMR by Hg (Skurnik et al., 2010).

The Arctic environment is a good illustration of the interconnectedness between environmental and human health—often termed One Health (Ruscio et al., 2015). One of the most thoroughly researched aspects of One Health in the Arctic is the Arctic Indigenous Peoples’ diet and its high content of contaminants, including heavy metals (AMAP, 2022). While the Indigenous diet is unquestionably the best source of health and nutrients in the Arctic, the positive impact of the diet has been questioned because of the content of toxic compounds, including heavy metals, ultimately leading to a framing of the diet as “The Arctic Dilemma”: weighing the benefits against the disadvantages of the Arctic Indigenous diet (Hansen, 1997). Hg and Cd are the primary heavy metals of concern in the Arctic diet stemming from the high intake of marine mammals (AMAP, 1998). In the animals consumed, Hg concentrations are highest in the liver, followed by the kidney, and then the muscle. However, in polar bears, the kidney has the highest concentrations. Cd is generally found in higher concentrations in the kidney, followed by the liver, and then the muscle (AMAP, 2003). Pb concentrations in the Inuit diet are generally low, but the intake of Pb has been shown to exceed tolerable daily intake when eating birds hunted with lead shots (Johansen et al., 2001, 2004). While heavy metals have been a primary focus in Arctic diet research, this aspect has not been considered in previous assessments of the gut microbiomes of Indigenous communities in the Arctic (Dubois et al., 2017; Girard et al., 2017).

In a previous study, we identified a diverse array of contaminant-resistance genes in Arctic environmental bacteria (Hauptmann et al., 2017). In this study, we aimed to study the potential response of the gut microbiota to heavy metals in the diet stemming from the environment. We used a multiomics approach comprising metatranscriptomics, 16S ribosomal (rRNA) gene amplicon data, and metagenome data to assess the following hypotheses:

- If heavy metals negatively impact the gut microbiota at the ecosystem level, we expect to see effects on the diversity, richness, and resilience of the gut microbiome ecosystems expressed through α- and β-diversity measures.
- If the heavy metals at the concentrations found in Arctic diets drive a change in the autochthonous gut microbiota toward an increase in already existing taxa with resistance to specific heavy metals, we expect to see a relatively higher fraction of resistant microorganisms as a response to a higher level of the metal in question.
- If heavy metals lead to the expression of autochthonous HMR genes, we expect a positive correlation between HMR gene expression levels and heavy metal concentrations.

## 2 Materials and methods

### 2.1 Participants

Study participants were self-enrolled volunteers. This study was granted ethical approval by the Government of Greenland on February 15, 2018 (file number 2018–2876, document number 7304874). Participants were allowed to enter the project after an oral presentation of the field of gut microbiome research and the current study’s theme, followed by a discussion in Kalaallisut (West Greenlandic dialect). The presentation was held in Danish and Kalaallisut and translated into the North Greenlandic dialect by a local translator with experience in collaborating with researchers. Participants were then given written consent forms, including information about the study, in Kalaallisut to sign before joining the project.

### 2.2 Fecal sample collection and processing

Four participants from northern Greenland, Avanersuaq, self-collected fecal samples once a month for 15 months from March 2018 to May 2019. Sampling required continuous coordination with each participant as well as the local healthcare center and was dependent on highly unpredictable infrastructure. Five samples were lost in the process (two samples from one participant and one each from the others), resulting in a total of 55 fecal samples. Samples for DNA extraction were collected using OMNIgene®•GUT kits (reference OM-200) (DNA genotek, Ottawa, Ontario, Canada), while samples for RNA extraction were collected in 3-ml RNA later® (Sigma–Aldrich, Merck, Darmstadt, Germany).

### 2.3 DNA and RNA extraction, library preparation, and sequencing

DNA was extracted using NucleoSpin® Soil (MACHEREY-NAGEL GmbH & Co. KG, Dueren, Germany). The concentration of DNA was checked using a fluorometer, and gel electrophoresis was used to check the integrity and purity of the samples (agarose gel concentration: 1%, voltage: 150 V, electrophoresis time: 40 min). Whole-genome library preparation and sequencing were performed according to BGI Genomics protocol SOP-EXC-J019, v.A1 (11 June 2018). In summary, 1 μg of genomic DNA was randomly fragmented using the Covaris® LE220 (Woodingdean, Brighton, UK). Fragment selection using Agencourt AMPure XP beads (Beckman Coulter, Inc., Brea, CA, USA) was performed on the disrupted sample magnetic beads to concentrate the sample bands at approximately 300–400 bp. Double-stranded DNA was end-repaired, and 3′ was adenylated to prepare a linker ligation reaction system. Sequencing on DNBSEQ-G400 was performed as described in the user manual (Wuhan MGI Tech Co. Ltd., 2019).

16S rRNA gene V4 amplicons were prepared and sequenced according to BGI Genomics SOP-MET-J006 v.A1 (20 November 2015) using a dual-index paired-end (PE) approach. Fusion primers were designed using P5 (5′ AATGATACGGCGACCACCGA 3′) and P7 (5′ CAAGCAGAAGACGGCATACGAGAT 3′) Illumina adapter sequences, an 8-nt index sequence, and V4 primers (515F-806R). Polymerase chain reaction (PCR) reaction mixture was 30-ng DNA, 4-ng PCR primer cocktail (515F-806R), and 25-ng PCR Master Mix (Phusion® High-Fidelity PCR Master Mix, New England Biolabs, Inc., Ipswich, MA, USA). The PCR program was as follows: initial denaturation at 98°C for 3 min, 30 cycles of denaturation at 98°C for 45 s, annealing at 55°C for 45 s, extension at 72°C for 45 s, and final extension at 72°C for 7 min. The PCR products were purified with Agencourt AMPure XP beads (Beckman Coulter, Inc., Brea, CA, USA). Sequencing on DNBSEQ-G400 was performed as described in the user manual (Wuhan MGI Tech Co. Ltd., 2019). The resulting data on 16S rRNA genes were sparse; the maximum number of reads per sample was 3,935 reads/sample.

RNA was extracted using the QIAGEN RNeasy PowerFecal Pro Kit (QIAGEN, Hilden, Germany). Library preparation for metatranscriptomic sequencing was performed according to BGI Genomics protocol SOP-SS-031, v.A0 (11 June 2018). The total RNA concentration, RNA integrity number (RIN), 23S/16S, and size were determined using an Agilent 2100 Bioanalyzer (Agilent RNA 6000 Nano Kit) (Agilent Technologies, Inc. Waldbronn, Germany). DNase I was used to degrade single and double-stranded DNA. rRNA was removed from the total RNA using QIAseq® FastSelect™—5S/16S/23S removal (QIAGEN, Hilden, Germany) during stranded RNA library preparation, and RNA molecules were fragmented into small pieces using fragmentation reagent before cDNA synthesis. First-strand cDNA was generated using random hexamer-primed reverse transcription, followed by a second-strand cDNA synthesis. The synthesized cDNA was subjected to end-repair and then was 3′ adenylated. Adapters were ligated to the ends of these 3′ adenylated cDNA fragments. The PCR products were purified with Agencourt AMPure XP beads (Beckman Coulter, Inc., Brea, CA, USA) and dissolved in EB solution. Finally, the double-stranded PCR products were heat-denatured and circularized using the splint oligo sequence. The single-stranded circle DNA (ssCir DNA) was generated as the final library. The library was amplified with phi29 to make DNA nanoball (DNB), and the DNBs were loaded into the patterned nanoarray for PE100 sequencing on BGISEQ (DNBseq platform). After sequencing, the raw reads were filtered, removing adaptor sequences, contamination, and low-quality reads from the raw reads. Sequencing on DNBSEQ-G400 was performed as described in the user manual (Wuhan MGI Tech Co. Ltd., 2019).

### 2.4 Heavy metal assessments

Hg, Cd, and Pb have been the primary heavy metals of concern in the Arctic diet stemming from the high intake of marine mammals and the use of lead shots (AMAP, 1998; Johansen et al., 2001, 2004). The dry weight concentrations of these three heavy metals were measured as follows: fecal samples were dried, and their dry weight was measured. Medico Kemiske Laboratorium, Vedbæk, Denmark, analyzed the total metal concentrations. Approximately 0.7 g of dry matter was digested in a 5-mL digestion solution containing 7.5 M HNO_3_, 1.2 M HCl, and 1-ppm rhodium (as an internal standard). The sample was microwave-digested at 120°C. Finally, the sample was diluted and analyzed using the ICP-MS (7700X ICP-MS system, Agilent Technologies, Santa Clara, CA, USA). The quantification was carried out using certified reference standards (PlasmaCAL Custom Standard, SPC Science, Quebec, Canada).

### 2.5 Dietary surveys

Participants were asked to complete a 1-week recall survey of their diets on the day of feces sampling. An overview of samples and metadata can be found in Supplementary Table S4. The data in this study are presented with a high degree of anonymity to respect the privacy and personal integrity of the participants in this project, who are members of a small community. Therefore, the sex of the participants and their detailed dietary results are not presented but are known to the research team.

Samples were assigned a local food score based on the diversity of a selected number of key local foods, namely, narwhal/beluga, polar bear, mattak (whale skin and blubber), seal blubber, intestines, dried meat/fish, and fermented foods. The participants were asked to answer “yes” or “no” to whether they had eaten the food in the past week. The number of “yes” corresponds to the score. These foods were selected as they are not eaten casually together with imported foods but rather are eaten on occasions when there has been an active choice of eating Indigenous foods. Therefore, they used these foods as a marker for a period when the participant consumed a high level of local foods.

### 2.6 16S rRNA gene amplicon bioinformatic analysis

Raw data were trimmed and quality-checked with Trimmomatic v.0.38 (Bolger et al., 2014) (SLIDINGWINDOW:4:15, MINLEN: 30) and was subsequently merged with VSEARCH v.2.14.2 (Rognes et al., 2016). Merged reads were processed using Qiime 2 v.2020.8 (Bolyen et al., 2019), denoising with deblur (Amir et al., 2017).

### 2.7 Metagenome bioinformatic analyses

The PE sequencing data from the metagenomic samples (*n* = 55) were collapsed for read pairs where read termini overlapped ≥11 base pairs using NGmerge (Gaspar, 2018). Next, collapsed and PE reads were mapped separately to the human reference genome (GRCh38) using BWA-mem (v.0.7.17) (Li and Durbin, 2009). Reads mapping to the human reference genome (mapping quality ≥1) were discarded from downstream analyses to ensure that metagenomic samples were devoid of sequencing data originating from the host. Each metagenomic sample was assembled individually using Metaspades (v.3.9.0, “-k 21,33,55,77”) (Nurk et al., 2017), followed by an open-reading-frame prediction using Prodigal (v.2.6.3, settings -p meta) (Hyatt et al., 2010). Only complete genes with a start and stop codon were retained for further analysis. The non-redundant gene catalog was constructed by sequence clustering of predicted genes, which grouped sequences with more than 95% identity and 90% coverage of the shorter sequence using CD-HIT (v.4.8.1) (Huang et al., 2010). The reads were then mapped to the non-redundant gene catalog with BWA-mem (v.0.7.16) (Li and Durbin, 2009) and filtered to retain reads mapping to genes with at least 95% sequence identity over the length of the read, counted (count matrix), and normalized to reads per kilobase per million to form a gene abundance matrix using in-house Python scripts. To calculate the corresponding expression of each gene, we mapped metatranscriptomic reads to the gene catalog using the same tools and sensitive cut-offs as described earlier.

To establish metagenomic species (MSPs), the count matrix derived by mapping metagenomic reads to the gene catalog was processed using MSPminer (v.2.0, default settings) (Oñate et al., 2019). This tool grouped the genes of the gene catalog into the MSP pan-genomes based on the defined core and accessory genes. Bacterial abundance profiles across samples for each MSP were calculated as a median transcript per million (TPM) using the 30 top representative core genes reported for each MSP using MSPminer.

Discovery and taxonomic annotation of bacterial species in the data were performed in the following steps: prior to binning, individually assembled metagenomic samples were filtered for contigs of a minimum of 2,000 base pairs long. The reads were mapped to all contigs with minimap2 (v.2.6, “-N 50”) (Li, 2018) and then filtered and sorted with Samtools (v.1.9, “-F 3584”) (Li et al., 2009). Contig abundance profiles were then calculated using the jgi_summarize_bam_contig_depths module from MetaBAT2 (v.2.10.2) (Kang et al., 2019) to produce a jgi-depth matrix with contigs abundances across all samples. The jgi-depth matrix was used as input to the metagenomic binner VAMB (v.3.1) (Nissen et al., 2021) that applies a deep-learning framework to cluster the metagenomic contigs into biological entities using the jgi-depths and tetranucleotide frequencies derived from input contigs. Bacterial metagenomic bins (metagenome-assembled genome [MAGs]) were identified using the lineage-wf of CheckM (v.1.1.2) (Parks et al., 2015), and near-complete (NC) bins with completeness of ≥90% and contamination ≤5% were retained for further taxonomic analysis. The taxonomy of each bacterial bin was determined using the *classify-wf* of GTDBK-TK (v.1.3.0) (Chaumeil et al., 2019), based on database release 95. To produce a set of representative MAGs, NC bins were then dereplicated at 99% average nucleotide identity (ANI) across 50% of the smaller genome using coverM (v.0.6.1) (Woodcroft and Newell, 2017) to yield 797 representative MAGs. Furthermore, coverage, RPKM, and TPM, for all representative MAGs, were calculated for each sample using coverM.

### 2.8 Annotation of heavy metal and antimicrobial resistance genes in metagenome data

To determine the fraction of reads mapping to HMR genes in each metagenome sample, forward reads were subsampled to the minimum number of reads across samples (32 million reads) using VSEARCH v.2.14.2 (--fastq_qmin 30) (Rognes et al., 2016) and were subsequently processed through MGmapper single end v.3.0 (Petersen et al., 2017) mapping against its homology reduced (threshold 0.8) metal resistance database. The full list of resulting HMR genes used to test for correlations with HMR gene read counts from whole-genome data can be found in Supplementary material S1. To characterize the prevalence and expression of microbially encoded HMR genes, we annotated the genes of MAGs using the BacMet database (Pal et al., 2014), composed of curated high-quality genes verified by experimental validation. Genes encoded by MAGs were predicted with Prodigal (v.2.6.3, settings -p meta) and then annotated using blastp (v.2.10.0) (Johnson et al., 2008) accepting only hits with >40% sequence identity and query coverage >30%. However, to identify the confident genomic islands of metal resistance, only bins with at least one gene annotated with >80% sequence identity and query coverage >80% to the BacMet database were retained. The AMR genes in MAGs were also annotated using the Comprehensive Antibiotic Resistance Database (CARD) (Alcock et al., 2020) (v.4.2.2) accepting only *strict* hits. Contigs encoding both metal and antibiotic resistance (co-resistance islands) included initial blast hits to the BacMet database (>40% sequence identity and query coverage >30%) and strict CARD hits.

### 2.9 Metatranscriptome bioinformatic analysis

Transcriptomic data was processed with SAMSA2 v.2.2.0 (Westreich et al., 2018). RefSeq (O’Leary et al., 2016) results from the SAMSA2 pipeline were used as input for downstream analysis of read counts of HMR genes. Genes with a total count across all samples <550 were excluded to have, on average, >10 counts per gene per sample across the 55 samples. For the principal component analysis (PCA) and probabilistic estimation of expression residuals (PEER) factors (Stegle et al., 2012), the remaining genes were VST-transformed using R-package DESeq2 v.1.26.0. Canonical correlation analysis (CCA) was carried out on the first seven principal components correlated to the individual by leaving out each sample, calculating the CCA, and projecting the sample onto the CCA space (leave-one-out) with the R-package CCA v.1.2.1. A CCA without leave-one-out is also shown. PEER factors were calculated using individual heavy metals and whether the samples were collected during the winter months (November through April) as covariates, using the R-package PEER v.1.0. The variance explained distributions were calculated using the R-package variancePartition v.1.16.1. Differential expression analysis with heavy metals, along with subsequent log2-fold change (FC) shrinkage, was carried out using the R-package DESeq2 v.1.26.0, utilizing the raw counts (without VST-transformation). For per-individual association analysis, only genes that were non-zero for more than half of the individual samples were used.

### 2.10 Statistics

Generic statistical analyses were conducted in RStudio 2021.09.2 build 382 (RStudio Team, 2022). Biological and environmental (BIOENV) analysis packages labdsv v.2.0.1 (Roberts, 2019) and vegan v.2.5.7 (Oksanen et al., 2018) were used to test for ASVs that were significantly associated with specific heavy metals by grouping samples by heavy metal concentration quartiles.

MicrobiomeSeq v.0.1 (Ssekagiri et al., 2017) together with phyloseq v.1.34.0 (McMurdie and Holmes, 2013) was used to evaluate correlations between individual taxa and heavy metal concentrations on a continuous scale.

## 3 Results and discussion

### 3.1 Fecal heavy metal concentrations reflect the intake of local foods

**Figure 1:**
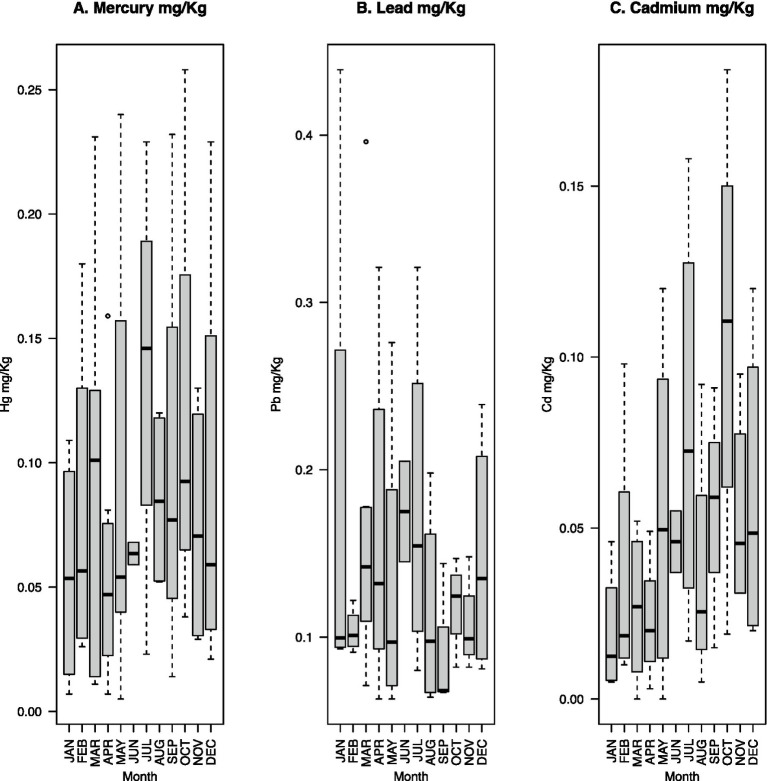
Boxplots of monthly fecal heavy metal concentrations (mg/kg dry weight) across all four participants (*n* = 55) for mercury (A), lead (B), and cadmium (C). The dashed lines indicate maximum and minimum values with outliers shown as dots outside of the lines; the bottom of the gray box indicates the 25th percentile; and the top of the gray box indicates the 75th percentile. The black line inside the gray box indicates the median.

The median fecal Hg content is highest in July (0.146 mg/kg) (Figure 1A). Pb has the highest median concentration in June (0.175 mg/kg), followed by July (Figure 1B). In contrast, Cd has the highest median concentration in October (0.1105 mg/kg), followed by July (Figure 1C). Dietary intake of Hg and Cd is expected to be highest in July, coinciding with the arrival of narwhals in northern Greenland (Rune Dietz, personal communication). This is reflected by the relatively high median Hg and Cd levels in July (Figures 1A,C).

**Figure 2:**
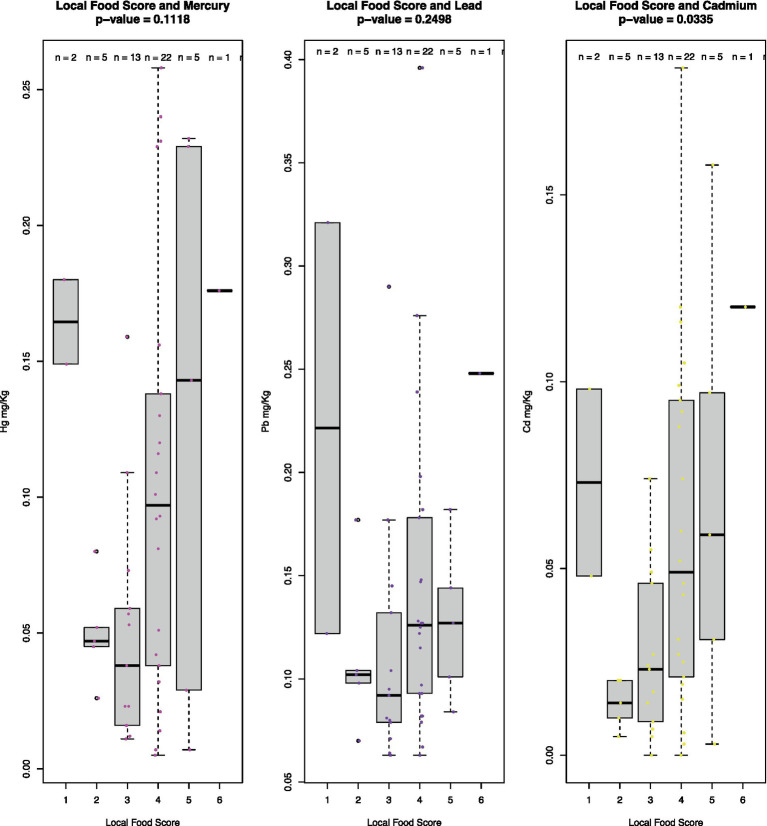
Boxplot of concentrations of Hg, Pb, and Cd (mg/kg dry weight) across samples stratified on local food score. Local food scores are based on the diversity of a selected number of key local foods (narwhal/beluga, polar bear, mattak [whale skin and blubber], seal blubber, intestines, dried meat/fish, and fermented foods). The number of these categories of foods eaten within a week from the sampling corresponds to the score. The top and the bottom of the gray boxes represent the 25th and 75th percentiles, respectively. Statistical significance indicated with *p*-values was determined using the Kruskall–Wallis rank sum test. The score ranges from 0 to 6 but 0 s were omitted because true 0 s could not be distinguished from false 0 s stemming from missing entries in dietary surveys.

It is expected that Hg and Cd levels correlate with the level of intake of local foods, which was true for Cd (*p* = 0.0335) (Figure 2). Hunting season for seabirds, which has been associated with intake of Pb, is between mid-October and April in northern Greenland. This does not seem to be reflected in the fecal concentrations of Pb, suggesting that the local diet is not a main source of Pb exposure, as also confirmed by the lack of association between Pb and the level of intake of local foods (Figure 2, *p* = 0.2498) and noted elsewhere (Johansen et al., 2004).

The median fecal Hg concentration of 0.07 mg/kg across all samples was more than double that of the concentrations observed in the fecal samples of two cohorts of American pregnant women, where medians were 0.03 and 0.028 mg/kg, respectively (Rothenberg et al., 2016, 2019). The mean concentration of Hg of 0.087 mg/kg across all samples was lower than that observed in feces in a previous study of 0.15 mg/kg (Rothenberg et al., 2016). In our data, the month with the highest concentration of Hg shows a median of 0.146 mg Hg/kg and a mean of 0.136 mg Hg/kg (Figure 1A). These values are notably higher than the median and mean concentrations in the previous studies mentioned above. The Hg concentrations found in these aforementioned studies were noted to be 5–10 times lower than previously observed levels based on hair samples (Rothenberg et al., 2016). Taken together, the Hg concentrations observed in the current study show fluctuations with concentrations that reach higher levels than previously described in studies focused on pregnant women. Cd concentrations detected in the present study (maximum 0.184 mg/kg, median 0.031 mg/kg, mean 0.0474 mg/kg dry weight) are notably lower compared to previously observed Cd concentrations in feces from people living in contaminated areas, which showed maximum levels of 4.49 mg/kg, a median of 0.28 mg/kg, and a mean of 0.54 mg/kg dry weight (Yabe et al., 2018). This illustrates the greater impact of environmentally sourced heavy metal contamination over dietary sourced contamination. Very high concentrations of fecal Pb have been detected among children living near a lead-zinc mine in Kabwe, Zambia, with levels reaching up to 2,252 mg/kg dry weight. The medians were reported approximately at 31.9 mg/kg dry weight, while the averages were approximately 90.6 mg/kg dry weight when comparing different sites (Yabe et al., 2018). The much lower Pb concentrations found in the present study, with a median of 0.122 mg/kg dry weight, a mean of 0.143 mg/kg dry weight, and a maximum of 0.439 mg/kg dry weight, do not reflect Pb pollution from the environment. The relatively low concentration of Pb might explain the low expression of Pb resistance genes (Supplementary Figure S1) and the fact that the Pb-specific resistance genes (*zntA* and *zraS*) were not found in any of the metagenome data. Consistent with previous findings (AMAP, 2003), we saw a correlation between Hg and Cd (Adj-*R*^2^ = 0.365), Hg and Pb are less correlated (Adj-*R*^2^ = 0.1285), and we observed no correlation between Pb and Cd (Adj-*R*^2^ = −0.014) (Supplementary Table S1; Supplementary Figure S2). The levels of the individual contaminants were not significantly correlated with the occurrence of apex predators in the diet, namely, polar bears, belugas, and narwhals (Supplementary Figure S3).

### 3.2 Hg, Cd, and Pb do not impact the gut microbiota at the ecological level

**Figure 3:**
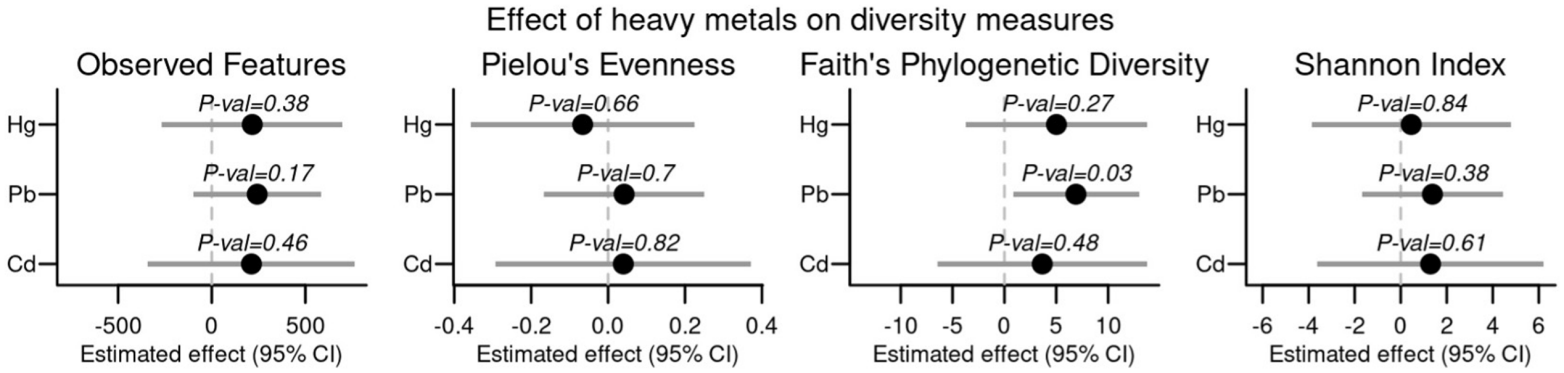
Effect and 95% confidence interval of the effect of heavy metal concentrations of mercury (Hg), lead (Pb), and cadmium (Cd) on four different diversity measures (observed features, Pielou’s evenness, Faith’s phylogenetic diversity, and Shannon index) calculated from 16S rRNA gene amplicon sequencing adjusted for individual.

No significant correlations were observed between α-diversity metrics (Observed Features, Pielou’s Evenness, Faith’s Phylogenetic Diversity, and Shannon Index) and the concentrations of Hg, Pb, or Cd (Figure 3). We calculated the average β-diversity (Bray–Curtis) distance between samples grouped per individual to see if participants with higher concentrations of either of the three heavy metals had higher average intra-individual β-diversity distance than participants with relatively low heavy metal load, which was not the case.

If heavy metals at the concentrations found in the northern Greenlandic diet drive gut microbiota changes at the ecosystem level, we would expect to see impacts on the diversity, richness, and resilience of these ecosystems expressed through α- and β-diversity measures as proposed in hypothesis i. We did not observe any significant impact on α-diversity with increasing levels of contaminants, as also found in other studies where Hg was found not to have any significant impact on human gut microbiota community structure *in vivo* and *in vitro* (Rothenberg et al., 2016; Guo et al., 2018). Furthermore, β-diversity dissimilarity distances within participants did not significantly increase with increasing levels of contaminants found for the participant, suggesting that ecosystem resilience is not influenced by the level of contamination found in the current study. Our findings align with previous studies showing no significant impact from heavy metals at the ecological level of the human gut, and this was supported further by the fact that there was no significant correlation between metagenome read counts for genes conferring HMR and the concentration of the corresponding metal. There was also no significant difference between the relative counts of metagenome reads mapping to HMR genes for Hg, Pb, and Cd between the current dataset and a Danish cohort (*n* = 62, manuscript in preparation).

### 3.3 Hg, Cd, and Pb have a relatively small influence on gene expression

PCA and CCA analyses based on the first seven principal components of the RNA expression data across all analyzed genes show that the samples cluster by individual (Supplementary Figures S4, S5). Individual is also one of the most important factors, explaining 27.3% of the transcription variance on average. After adjusting for individuals and the first three PEER factors, the heavy metal concentrations explain a relatively small amount of variance for the majority of genes, and the remaining mean residual variance is 46.7% (Supplementary Figures S4, S7).

Association analysis of heavy metals predicting gene expression across all individuals showed five genes significantly associated with Pb and eight genes with Hg (Supplementary Table S2). No gene expression was significantly associated with Cd. Separate association analysis within each individual showed an expression of “nitrate ABC transporter permease” being significantly associated with Hg (L2FC = 16.54, BH-adj *p*-value = 3.67E−06) and Cd (L2FC = 16.08, BH-adj *p*-value = 1.68E−03). The *p*-value quantile−quantile (QQ)-plots and variance stabilizing transformation (VST)-transformed counts plotted against both Hg and Cd are shown in Supplementary Figure S7. The highest log_2_FC for Pb is lactose ABC transporter permease (log_2_FC 7.54, *p*_adj_ = 7.80E−03, Supplementary Table S2). Bacterial adenosine triphosphate (ATP)-binding cassette (ABC) systems are, among many other functions, exporters of toxic molecules (Davidson et al., 2008), including heavy metal detoxification (Prévéral et al., 2009). The importance of ABC systems in heavy metal detoxification is further supported in the current data in that nitrate ABC transporter permease was also significant for Hg and Cd separate association analysis per individual. The highest log_2_FC for Hg is a conserved histidine α-helical domain (CHAD)-containing protein (log_2_FC 9.95, *p*_adj_ = 1.74E−02). CHAD is an uncharacterized domain (Letunic et al., 2021) that might participate in metal chelation (Iyer and Aravind, 2002; Letunic et al., 2021).

### 3.4 Heavy metal-resistant strains of the gut microbiome

**Figure 4:**
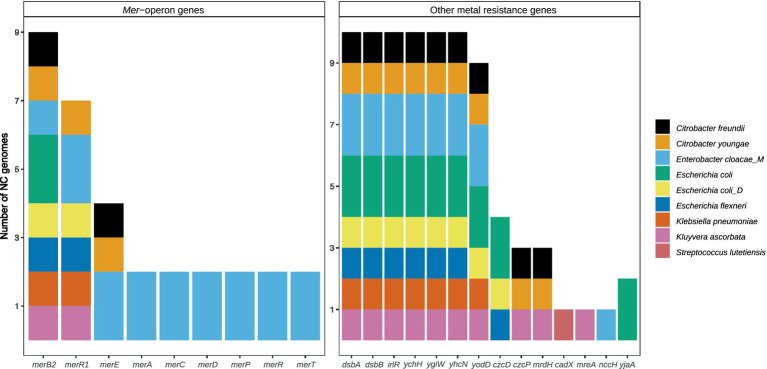
The number of near-complete (NC) metagenome-assembled genomes (MAGs) containing individual Hg, Cd, and Pb resistance genes. *Mer*-operon genes to the left and other resistance genes to the right.

To obtain further insights into the heavy metal resistome, metagenome data were binned into MAGs, resulting in 3,455 near-complete (NC) genomes of 8,726 bins (>1 Mbp). These NC MAGs were dereplicated into 797 genomes, representing a median of 80% of reads from each sample on average. Through annotation to BacMet and strict filtering (>80% identity, >80% coverage), genomes with HMR genes were identified (Figure 4).

There were nine MAGs carrying HMR genes (HMR MAGs), including *Citrobacter freundii*, *Citrobacter youngae*, *Enterobacter cloacae*, *Escherichia coli*, *Escherichia coli* (type D), *Escherichia flexneri*, *Klebsiella pneumoniae*, and *Kluyvera ascorbata*, which are all *Enterobacteriaceae*, and finally one Gram-positive *Streptococcus lutetiensis* (Figure 4). None of the heavy metal-resistant MAGs were identified as significantly correlating with concentrations of any of the heavy metals using three different methods (BIOENV, ACOMBC, and microbiomeSeq, Supplementary Table S3). No taxa were identified as having the same significant association with a certain heavy metal by all three methods.

### 3.5 Expression of the toxicity-reducing *mer*-operon in the human gut

**Figure 5:**
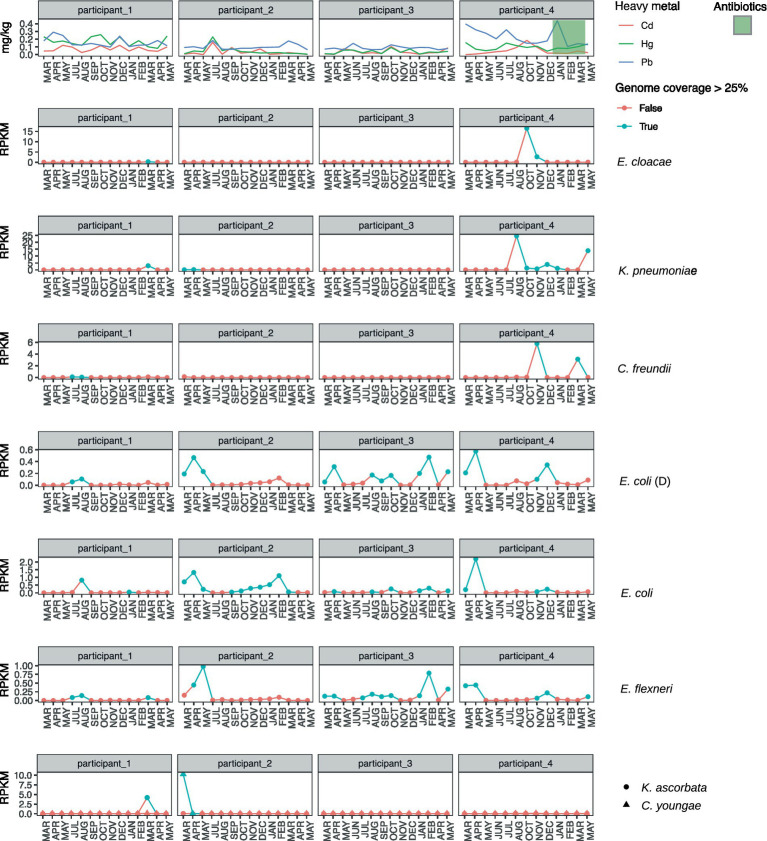
Distribution of HMR MAGs across participants (columns) and months (*x*-axes). The longitudinal concentration profiles of cadmium (Cd), mercury (Hg), and lead (Pb) are displayed for participants (1–4) in the first row. The subsequent rows, one for each species, display the reads per kilobase million (RPKM) profiles of *Enterobacter cloacae*, *Klebsiella pneumonia*, *Citrobacter freundii*, *Escherichia coli* (type D), *E. coli*, *Escherichia Flexneri*, and *Kluyvera ascorbata* together with *C. youngae* (indicated by different shapes). Detection of a specific bacteria at a given time point (month) is determined by whole-genome sequence coverage with at least >25% of the genome covered by a read (colored blue) or (red) if not detected. Participant 4 was the only one treated with antibiotics, which happened over three consecutive months and is indicated by a green-colored background in the first row.

**Figure 6:**
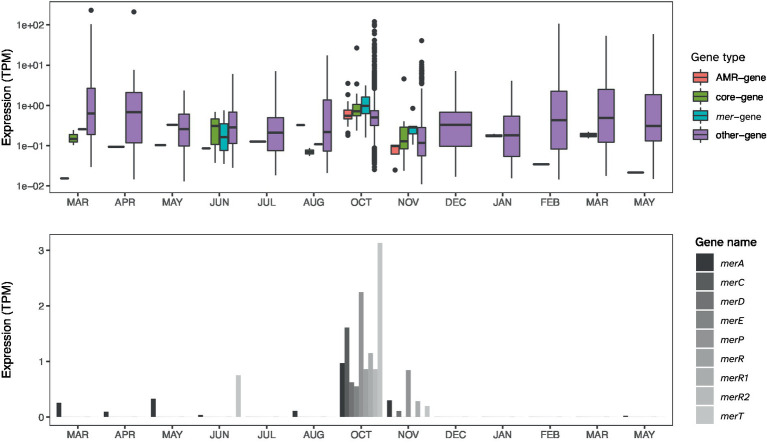
In the upper panel, the expression levels (TPM) of *Enterobacter cloacae* in Participant 4 of *mer*-genes, core genes, and remaining coding genes. Gene groups of *E. cloacae* were defined by MSP-miner as described in the Methods section. The lower panel shows the expression level of each of the *mer*-genes.

The *E. cloacae* identified among the HMR MAGs is distinguished by being the only HMR MAG with a comprehensive *mer*-operon, including *merACDEPRR1R2T* (Figure 4). The identified *E. cloacae* has the potential to reduce mercury toxicity in the gut through MerA, mercuric ion reductase, the key detoxification enzyme that transforms Hg into its volatile monoatomic vapor Hg(0), rendering it more volatile and less reactive (Barkay et al., 2003). The metatranscriptomic data showed that the samples in which *E. cloacae* was identified coincided with high levels of *mer*-gene expression exceeding that of other gene expression (Figures 5, 6). These results are relevant in light of the increasing attention that is being given to the search for bacterial strains with the potential to reduce heavy metal toxicity in the human gut and also beyond well-known probiotic strains (Duan et al., 2020). A recent review highlighted that a central question remains unanswered: which bacterial strains are responsible for the detoxification of heavy metals in the gut (Ghosh et al., 2023)? Through combining metagenomics and metatranscriptomics, we were able to add new relevant insights by showing an *E. cloacae* strain, which is actively expressing *mer*-genes, including toxicity reducing MerA (Figure 6) and its distribution across time (Figure 5).

In contrast to the *mer*-genes, the genes *hgcA* and *hgcB* methylate mercury, which makes it more bioavailable and therefore also more toxic (Parks et al., 2013). These genes are relatively rare (Podar et al., 2015) and were not found in any of the 55 metagenomic samples of this study, supporting results from previous studies (Zhou et al., 2011; Gilmour et al., 2013; Podar et al., 2015; Rothenberg et al., 2016). This indicates that the studied microbiomes show genetic potential for reducing the toxicity of mercury but not for increasing it.

The distribution of HMR MAGs (Figure 5) shows that the HMR *E. cloacae* is present in only three samples, which happen to be two consecutive samples from participant 4 and a low abundance in one sample from participant 1. The pattern in which *E. cloacae* is present in two consecutive months suggests either a colonization event with an allochthonous strain or a sudden increase in abundance of a very low-abundant strain. *E. cloacae* in the human gut has been linked to a Bacteroides enterotype (Raymond et al., 2016), often associated with a diet rich in meat and fat (Arumugam et al., 2011; Wu et al., 2011). In our data, the sample in which *E. cloacae* was first detected in participant 4 is distinguished by being the only sample among 13 monthly samples where the individual consumed local meats every day in the week up to the sampling. The presence of *E. cloacae* might be due to the coincidental interplay between heavy metals being correlated with local food intake and local foods in this particular context being rich in animal fat and protein, thereby enhancing the chance of successful colonization with *E. cloacae*. Guo et al. (2018) showed an instance in which protein supplementation significantly increased demethylation and hypothesized that protein might enhance monomethyl mercury (MMHg) degradation due to syntropic interactions. Whether the protein- and fat-rich Arctic Indigenous diet increases the likelihood of colonization with *mer-*carrying *E. cloacae* is an interesting hypothesis to be tested. There are ongoing efforts that encourage a more nuanced understanding of Arctic Indigenous foods that take into consideration; for instance, the mitigating effect of the nutrient-rich foods, including the role of selenium in the Arctic diet as a protectant against contaminants (Laird et al., 2013; Liu et al., 2019). Selenium, which the Inuit diet is rich in (Hansen et al., 2004), increases the excretion of Hg (Liu et al., 2019). Furthermore, carbohydrates, which have only become a common dietary component in the Arctic after colonization, can inhibit demethylation (Lu et al., 2017), also by the human gut microbiome as well (Guo et al., 2018). Our study adds to the argument that it is relevant to investigate further how the Arctic Indigenous diet has a protective effect against contaminants, for example, by supporting detoxification through the demethylation of Hg.

### 3.6 Antibiotic-resistant strains in the gut are also resistant to heavy metals

**Figure 7:**
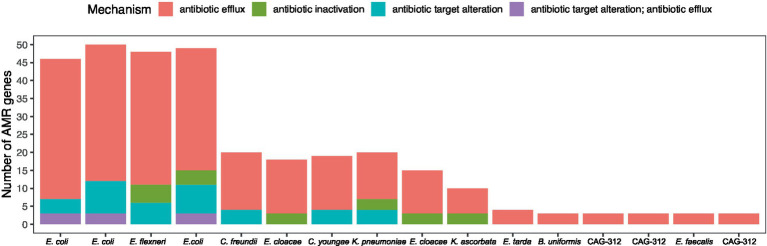
Antimicrobial resistance (AMR) annotation (strict) with CARD. Displayed is the total number of AMR genes for individual microbial genomes that we identified as having AMR. Genes are colored according to their CARD mechanism.

Ten of the 797 dereplicated MAGs, all belonging to the family *Enterobacteriaceae*, had more than one type of AMR mechanism and were also the 10 MAGs with the highest number of AMR genes (Figure 7). Remarkably, these 10 AMR MAGs were also resistant to HM (Figure 4), showing an almost complete overlap between strains with resistance to heavy metals (HM) and antimicrobials (AMs). Early culture-based studies established a correlation between Hg resistance and AMR in the human gut microbiome (Edlund et al., 1996). Since then, a comprehensive omics-based assessment of environmental and clinical bacterial genomes showed that 17% of all studied genomes have co-occurrence of HMR and AMR (Pal et al., 2015). The same authors more recently reviewed the knowledge gaps in this field, listing no gut microbiome studies and emphasizing the need for data representing genome-level understanding within complex environments (Pal et al., 2017), which is what the current study adds. Our data show that in the human gut, AMR is strongly associated with HMR at the genome level. Specifically, 100% of the strains in the top 10 with the highest number of AMR genes, including those with more than one type of AMR, are also HMR resistant (Figures 4, 7; Table 1). Additionally, we show that all these HMR-AMR MAGs are co-resistant through the co-occurrence of AMR and HMR genes on the same genetic element (Table 1). Finally, our chronological sampling design allows us to demonstrate that such AMR-HMR strains are found in all participants and in varying abundance through time often falling to undetectable levels (Figure 5), which has implications for snapshot studies that might miss important information on the gut microbiome’s capacity for co-resistance. AMR is one of the top 10 global public health threats facing humanity (WHO, 2021). The importance of heavy metals as a driver for the dissemination of AMR genes in the environment is well recognized (Czatzkowska et al., 2022), but its impact as a consequence of heavy metal pollution in the Arctic is less understood. This is evident in the literature on the prevalence of antibiotic-resistance genes in the Arctic environment (Tan et al., 2018), which does not touch upon the co-occurrence of AMR and HMR, despite the well-established contamination with heavy metals in Arctic environments. This points to an important knowledge gap that might easily be bridged through interdisciplinary conversations.

**Table 1:** Co-resistance in metagenome-assembled genomes (MAGs) showing co-occurrence of antimicrobial resistance (AMR) and heavy metal resistance (HMR) genes on contigs.

MAG species	Contig	AMR genes and proteins	HMR genes and proteins
*Kluyvera ascorbata*	1	*ampH*	*merR1*
	2	*H-NS*	*ychH*
*Enterobacter cloacae*	1	*baeR*	*yodD*
	2	*acrA, adeF*	*merR1*
	3	*H-NS*	*dsbB, ychH*
	4	*CRP*	*irlR, yhcN,*
*Citrobacter freundii*	1	*cpxA*	*dsbA*
	2	*marA, QnrB67, H-NS*	*merE, ychH*
	3	*CRP, msbA*	*czcP, dsbA*
*Escherichia coli* (type D)	1	*AcrF, AcrE, AcrS, bacA, TolC*	*yhcN, ygiW*
	2	*H-NS*	*merR1, ychH, dsbB*
	3	*msbA, mdfA*	*czcD, yhcN*
	4	*mdtP, mdtO, mdtN, eptA, ampC, soxS, soxR*	*merB2, yhcN*
*E. coli*	1	*AcrF, AcrE, AcrS, bacA, TolC KpnH, emrA, emrR*	*dsbA, yhcN, ygiW*
	2	*emrE*	*yodD, irlR*
	3	*soxR, soxS*	*merB2, yjaA*
	4	*msBa, mdfA*	*yhcN, czcD*
	5	*H-NS*	*dsbB, ychH*
	6	*kdpE, acrA, acrB, ampH, acrR*	*irlR*
*E. coli*	1	*ampH, acrB, acrA, kdpE, acrR*	*irlR*
	2	*AcrS, AcrE, AcrF*	*yhcN*
	3	*H-NS, mdtH*	*ychH, dsbB*
	4	*bacA, TolC*	*ygiW*
	5	*mdtP, mdtO, mdtN, eptA, ampC, soxS, soxR*	*yjaA, merB2, yhcN*
	6	*emrE*	*yodD, irlR*
*Citrobacter youngae*	1	*marA, QnrB8, H-NS, mdtG, PmrF, GlpT, marR*	*yodD, dsbB, merE, ychH*
	2	*KpnH, emrR*	*mrdH*
	3	*cpxA*	*dsbA*
	4	*CMY-17*	*yhcN*
	5	*soxS*	*merB2*
	6	*CRP, UhpT*	*czcP*
	7	*ampH, acrB, acrA, kdpE, mdfA*	*irlR*
	8	*mdtB, baeR, msbA*	*dsbA*
*Escherichia flexneri*	1	*AcrS, AcrE, AcrF*	*yhcN*
	2	*mdtM, FosA2*	*yhcN*
	3	*ampC, eptA, mdtN, mdtO, mdtP, soxR, soxS*	*merB2*
	4	*mdtG, mdtH*	*ychH, dsbB, merR1*
	5	*TolC, bacA*	*ygiW, irlR*
*Klebsiella pneumoniae*	1	*acrA*	*merR1*
	2	*CRP, EF-Tu*	*yhcN*
	3	*H-NS*	*merR1, ychH*

Based on contigs from metagenome assembly using Metaspades v.3.9.0 for each MAG, it was studied whether HMR genes co-occurred on contigs with AMR genes. For each MAG, the contigs with co-occurrence of HMR and AMR are listed.

AMR, antimicrobial resistance; HMR, heavy metal resistance; MAG, metagenome-assembled genome.

In conclusion, the concentrations of heavy metals—Hg, Cd, and Pb—at the concentrations found in the present study did not significantly impact α- and β-diversity measures. Therefore, they appear not to influence the gut microbiota at the ecological level. This aligns with previous studies and was further supported by the lack of correlation between metagenome read counts for genes conferring HMR and the concentration of the corresponding metal. In addition, Hg, Cd, and Pb concentrations only had small effects on gene expression. While the diets of Arctic Indigenous Peoples are often discussed in the context of contaminants, the current results on the gut microbiota suggest that heavy metals should not be emphasized as a significant factor. It appears that heavy metals are not a strong confounding factor in the study of Arctic Indigenous Peoples’ microbiomes. Among almost 800 MAGs, nine heavy metal-resistant strains were identified. Only one of the identified heavy metal-resistant strains, *E. cloacae*, harbored the *mer*-operon, which was actively expressed and, therefore, is likely to render Hg less toxic to the host. Our results support previous studies showing the human gut microbiome as a site for demethylation, causing a lessening of the mercury toxicity rather than one for Hg methylation.

## Data Availability

The datasets presented in this study can be found in online repositories. The names of the repository/repositories and accession number(s) can be found at: https://www.ebi.ac.uk/ena, ENA:PRJEB59239.
