# Supplementary material for: Gut heavy metal and antibiotic resistome of humans living in the high Arctic
Source: Front Microbiol. 2024 Oct 30;15:1493803. doi: 10.3389/fmicb.2024.1493803 (PMC11557323; doi:10.3389/fmicb.2024.1493803)
Supplement: Supplementary file 1 [file Data_Sheet_1.PDF]

Table S3
BIOENV, ANCOM-BC and microbiomeSeq results

The table lists for each of the three methods the identified species of Bacteria with a significant correlation to a heavy metal and in brackets the heavy metal to which it was found to correlate as well as the direction of correlation, +: positive correlation, -: negative correlation.

ASV Identity (correlated heavy metal and direction of correlation)
microbiomeSeq
Roseburia faecis (Cd+, Pb-)
Oscillospira sp. (Cd-, Hg-)
Bacteroides sp (Pb+)
Blautia sp (Hg+, Pb+)
Bacteroides sp (Cd+)
Blautia sp (Hg-)
Faecalibacterium prausnitzii (Cd+)
Bacteroides sp (Pb+)
Sutterella sp (Pb-)
Bacteroides sp (Pb+)
Lachnospiraceae (F) (Cd+, Pb-)
Sutterella sp (Hg-, Pb-)
Bifidobacterium adolescentis (Hg-)
Bifidobacterium adolescentis (Cd-, Hg-, Pb+)
Phascolarctobacterium sp (Pb+)
Blautia sp (Hg-)
ANCOM-BC
Dorea sp (Cd+)
Blautia sp (Hg+)
Sutterella sp (Pb+)
Acidaminococcus sp (Pb+)
Ruminococcus bromii (Hg+)
BIOENV quartiles
Oscillospira sp. (Hg-)
Blautia sp. (Pb+)
Eubacterium biforme (Pb+)
Lachnospiraceae genus unknown (Pb-)
Ruminococcus sp. (Pb+)
Bacteroides sp (Cd+)
Bacteroides sp (Cd+)
Bacteroides sp (Cd+)
Blautia sp. (Cd+)
Blautia sp. (Cd+)
Bacteroides caccae (Cd+)
Bacteroides sp (Cd-)
Bilophila sp (Cd+)
Parabacteroides sp (Cd+)
FAMILY Coriobacteriales (Cd+)
Prevotella copri (Cd+)
Bacteroides caccae (Cd+)
Lachnospiraceae genus unknown (Cd+)
ORDER RF32 (Cd+)
Bacteroides sp (Cd+)
ORDER RF32 (Cd+)
Prevotella copri (Cd+)
Sutterella sp (Cd+)
