# Supplementary material for: Gut heavy metal and antibiotic resistome of humans living in the high Arctic
Source: Front Microbiol. 2024 Oct 30;15:1493803. doi: 10.3389/fmicb.2024.1493803 (PMC11557323; doi:10.3389/fmicb.2024.1493803)
Supplement: Supplementary file 2 [file Data_Sheet_2.DOCX]

**

*Figure S1. A: Mercury (Hg), lead (Pb) and cadmium (Cd) concentrations in mg/Kg across all fecal samples (n=55). B. Counts of transcriptome reads for Hg resistance genes, Pb resistance genes and Cd resistance genes respectively normalized to total read count per sample. C: Counts of metagenome reads for Hg resistance genes, Pb resistance genes and Cd resistance genes respectively normalized to total read count per sample. Resistance genes for each contaminant are listed in Supplementary Document 3.*


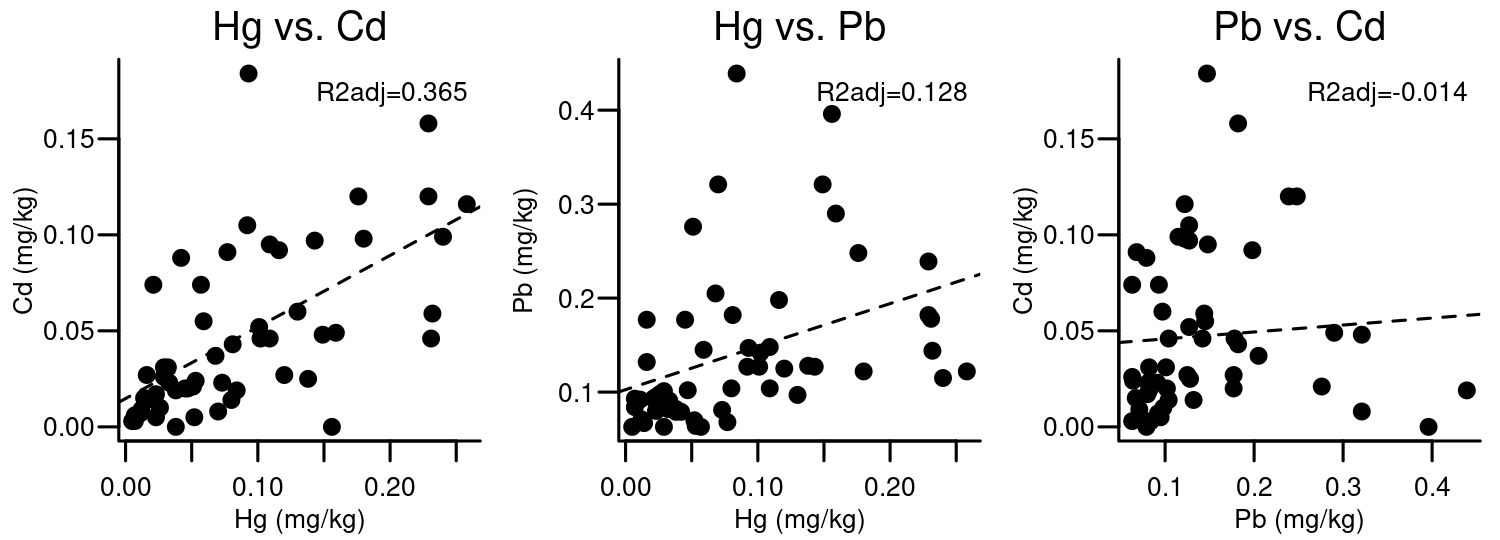
*Figure S2. Correlation between sample mercury (Hg), cadmium (Cd), and lead (Pb). Adjusted R2 of Hg predicting Cd or Pb and Pb predicting Cd reported in top right corner.*

**

*Figure S3. Fecal heavy metal concentrations (mg/Kg dry weight) of cadmium, mercury and lead respectively, across samples with or without consumption of apex predators.*


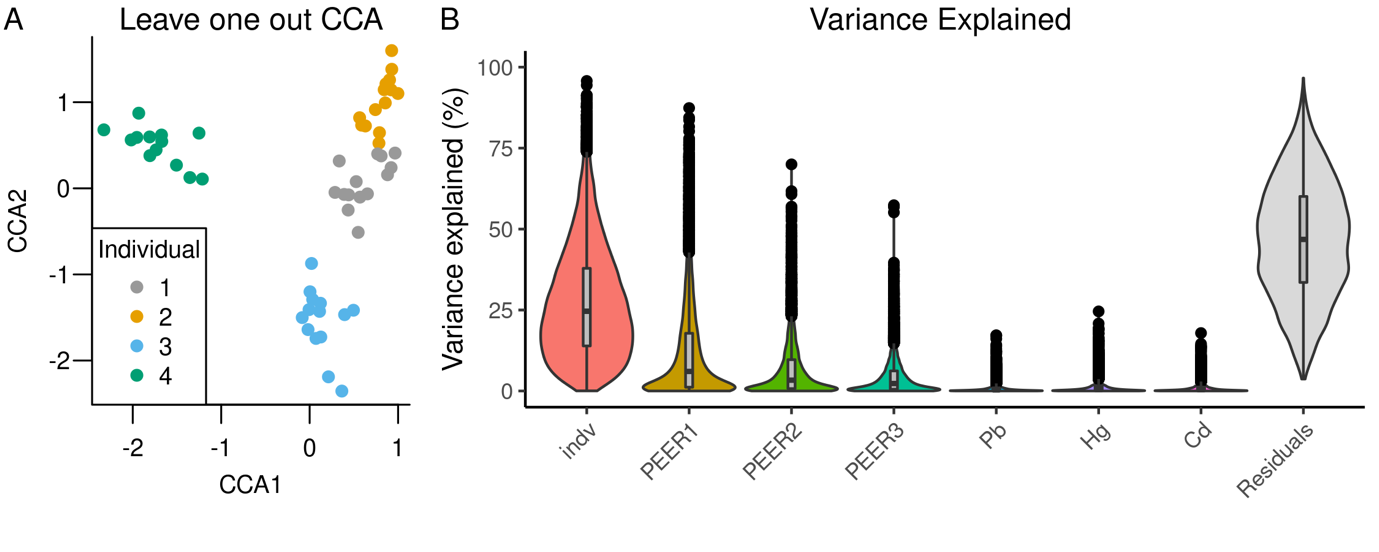
*Figure S4. A Leave one out CCA of the first 7 gene expression principal components correlated with individuals showing clear clustering of samples in each individual. B Variance explained distributions for the first 3 PEER factors, heavy metals and residuals. The mean variance explained by individual is 27.3% and the mean residual variance is 46.7% after modelling out individual, the first 3 PEER factors, and heavy metal content.*


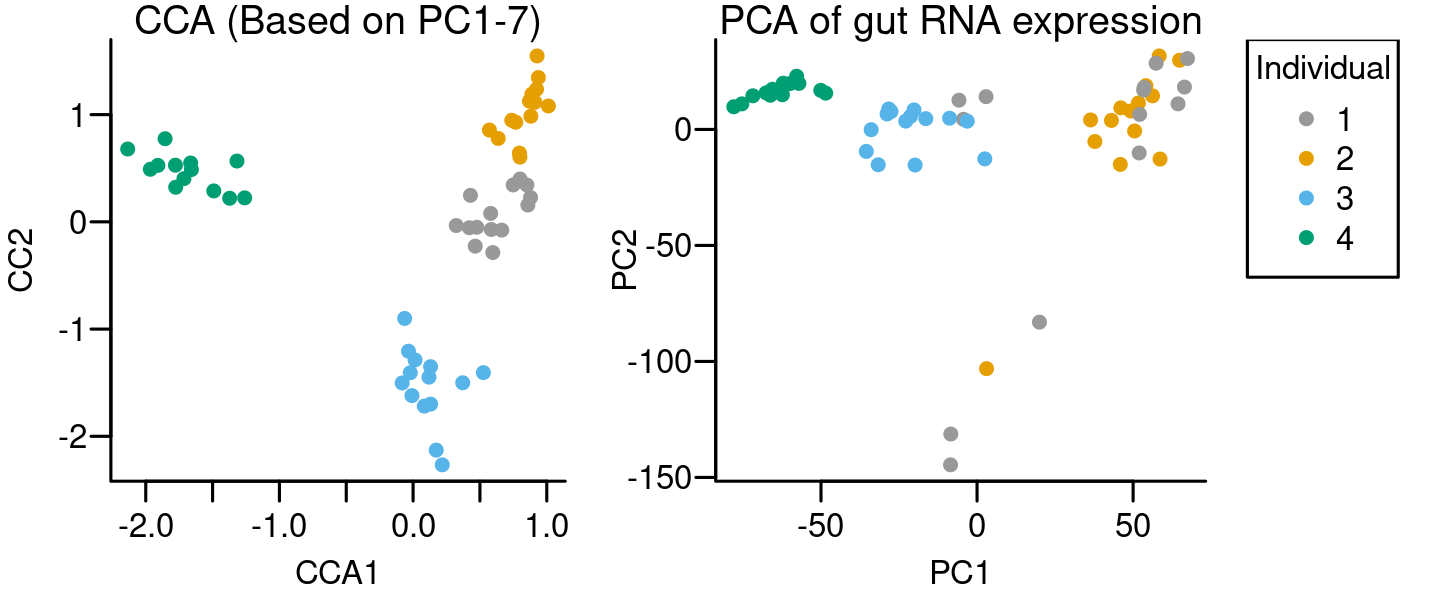
*Figure S5. Left: Standard CCA correlated with individuals based on the first seven principal components of the gut RNA expression. Right: The first two principal components colored by individual.*


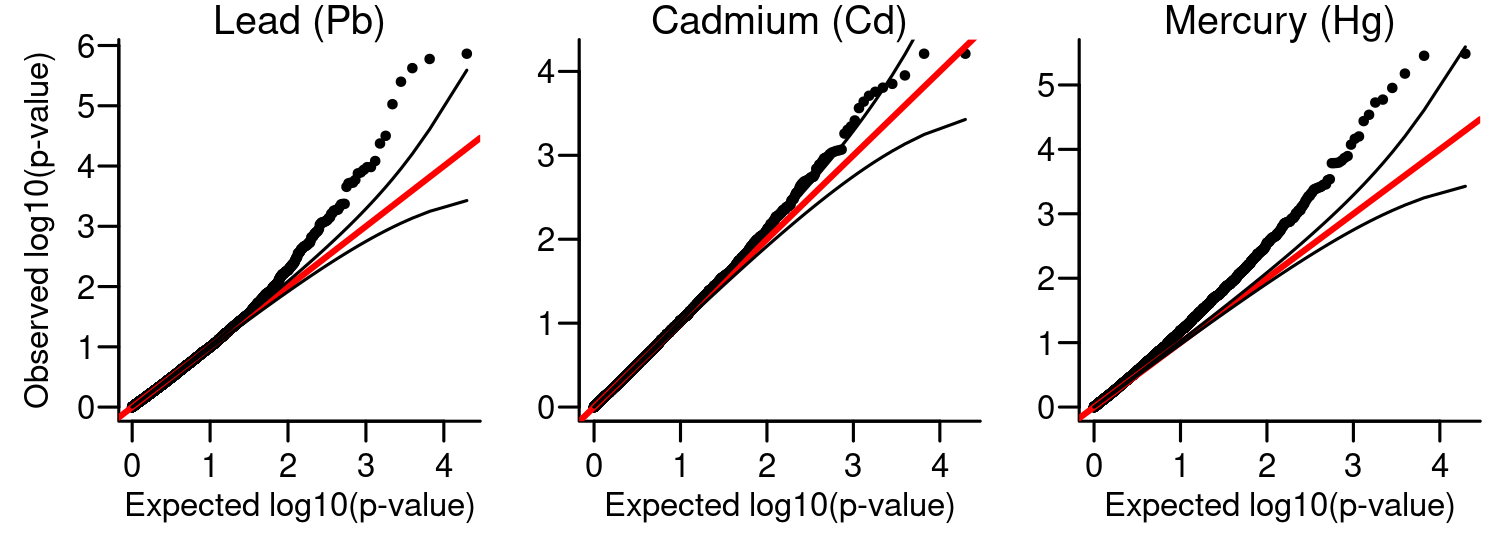
*Figure S6. P-value QQ-plots of gene expression association analysis with heavy metals adjusted for individual, the first 3 PEER factors, individual, and whether the sample is from a winter month or not.*


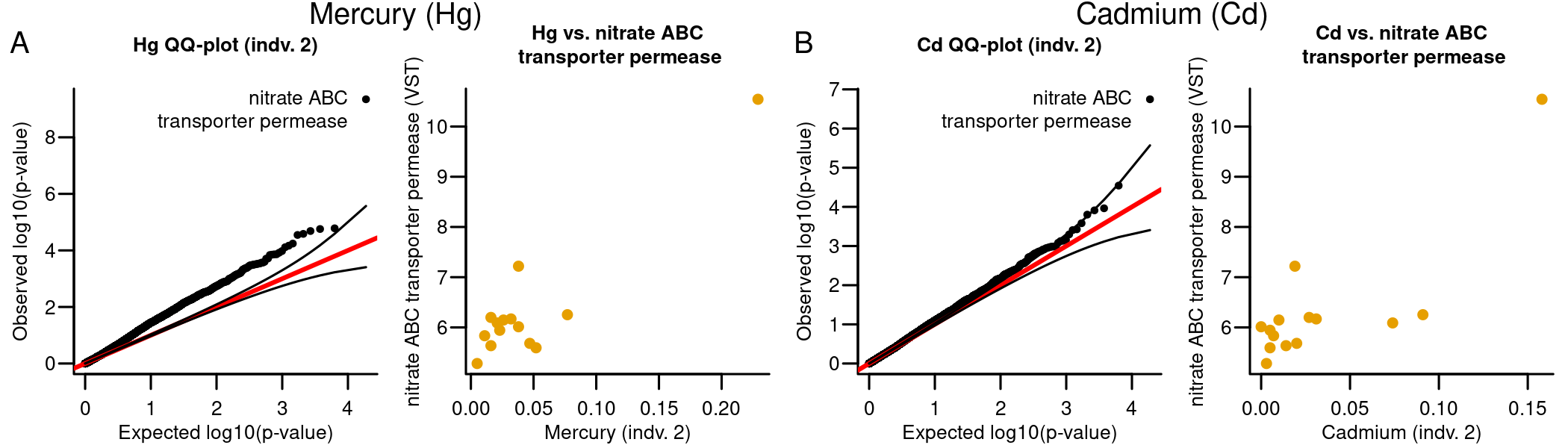


*Figure S7. Individual 2 association analysis with mercury (A) and cadmium (B). P-value QQ plots show relatively well-controlled test-statistics and the VST transformed counts of nitrate ABC transporter permease are plotted against both mercury (A) and cadmium (B) showing a linear correlation.*
