# Supplementary material for: Gut heavy metal and antibiotic resistome of humans living in the high Arctic
Source: Front Microbiol. 2024 Oct 30;15:1493803. doi: 10.3389/fmicb.2024.1493803 (PMC11557323; doi:10.3389/fmicb.2024.1493803)
Supplement: Supplementary file 3 [file Data_Sheet_3.docx]

Document S1: list of heavy metal resistance genes used to test for correlations with heavy metal resistance gene read counts from whole genome data

Mercury

merR1_BAB62429.1

merH_ACC43904.1

merB_AAC38230.1

merD_14_X03405

merT_11_AM048832

merC_11_AF071413

merR_AFB71197.1

merT_16_X73112

dsbA_AAC43530.1

merA_BAA20337.1

dsbA_AAC43533.1

merR_6_GQ293500

merA_AFB71200.1

smtA_CAA45873.1

merD_1_L29404

merT_AAA98244.1

merR_AAA98241.1

dsbA_AAA82614.1

dsbA_AAC43524.1

merA_AAA88368.1

merT_CAC69249.1

merA_CAC69251.1

merC_BAA14138.2

merT_AEJ04518.1

merP_AFB71199.1

merB2_BAA89007.1

merE_AAA88371.1

merP_BAA20336.1

merD_AAA88370.1

dsbA_BAE77448.1

merP_10_Z00027

dsbA_AAC43531.1

merD_12_AJ302764

dsbA_AAC43527.1

merP_AAC38232.1

dsbA_AAC43534.1

merD_CAI30248.1

merR_CAC69248.1

merD_CAA77324.1

dsbA_AAC43523.1

merT_BAA20335.1

merR2_BAA36431.1

merE_KZX56692.1

merT_4_AY033653

merC_5_GQ293501

merB3_BAA89005.2

merP_9_Y11706

merT_AAA98222.1

merB3_BAB62428.1

merR_12_AJ251517

merR_AAA98221.1

merR2_AAD25959.1

merP_AEJ04517.1

dsbA_AAC43522.1

merP_2_GQ293501

smtA_ABB57320.1

merT_KMP15553.1

merB1_AAA83978.1

merP_AAA98223.1

merB1_BAB62436.1

merB1_BAA82061.2

dsbA_AAC43526.1

merT_2_GQ293501

merT_AAC38231.1

dsbA_AAC43525.1

merF_ABA26000.1

merC_AAC38233.1

merR_AEJ04519.1

merR2_AAC38229.1

merA_AAA98245.1

merR_2_L20693

merB_BAA20338.2

dsbA_AAC43529.1

merP_5_AF071413

merT_9_Y11706

merD_AAC38221.1

merR_5_AY033653

merB_BAB47641.1

dsbA_AAC43519.1

merP_4_AY033653

merE_CAA77325.1

merD_BAA36433.1

dsbA_AAC43521.1

merD_11_X73112

merP_11_X73112

merT_CAA70196.1

dsbB_AAA23711.1

dsbB_AAA24220.1

dsbA_AAC43528.1

PA0320_AAG03709.1

merR1_BAA20334.1

merA_AAA98224.1

merB_2_U77087

merA_BAB62433.1

dsbA_AAC43535.1

merP_BAB62432.1

merD_AAC38235.1

dsbA_AAC43520.1

merA_AAC38220.1

dsbB_BAA36032.2

merD_4_GQ293501

merB_AAA88369.1

dsbA_AAC43532.1

merP_CAC69250.1

merT-P_AFB71198.1

merG_BAA23166.1

Lead

nmtR_CCP46571.1

pbrD_ABF12808.1

pbrR_ADJ63118.1

zraS/hydG_AAA24003.1

pbrT_CAI47902.1

cadC_AAB59153.1

pbrB/pbrC_CAI11270.1

zraS/hydG_BAE77316.1

pbrA_CAI11271.1

zntA/yhhO_BAE77824.1

Cadmium

czcB_KJ680225.1

nmtR_CCP46571.1

czcA_ABF12839.1

yjaA_BAE78013.1

ygiW_BAE77080.1

CzrCBA_EU527967.1

yhcN_AAA58040.1

cadA/yvgW_AAD07839.1

mdrL/yfmO_CAB43715.1

dsbA_AAC43530.1

dsbA_AAC43533.1

dsbA_AAA82614.1

czcC_CAA50525.2

dsbA_AAC43524.1

cznA_CBG40022.1

yhcN_BAE77281.1

czcB_CAA67083.1

nccN_AAA65107.1

dsbA_BAE77448.1

mntH/yfeP_BAA16262.1

nccX_AAA65102.1

nccC_AAA65104.1

dsbA_AAC43531.1

dsbA_AAC43527.1

dsbA_AAC43534.1

irlR_CAH38501.1

fieF/yiip_BAE77395.1

czcA_CAA67084.1

dsbA_AAC43523.1

czcC_ABF12841.1

cadB_2_AY048756

dsbA_AAC43522.1

actS_AAB38420.1

zinT/yodA_BAE76561.1

cznB_CBG40021.1

cadR_AAK48830.1

fpvA_AAG05786.1

dsbA_AAC43526.1

frnE_AAF10238.1

yodD_BAE76554.1

dsbA_AAC43525.1

czcE_ABF12835.1

nccA_AAA65106.1

czcR_CAA67086.1

czcD_ABF12838.1

cadA_1_AM990992

czc_KJ680225.1

mrdH_AAN68576.1

nccB_AAA65105.1

czrC_KF593810.1

cadB_1_U74623

dsbA_AAC43529.1

zupT/ygiE_BAE77096.1

czrA_CAB56471.1

cadX_ABS57010.1

dmeF_ABF07084.1

czcB_ABF12840.1

czcS_ABF12836.1

irlS_AAB92483.1

cadA/yvgW_AAA93043.1

dsbA_AAC43519.1

czrB_AB016431

dsbA_AAC43521.1

fpvA_AAA25819.1

dsbB_AAA23711.1

dsbB_AAA24220.1

dsbA_AAC43528.1

PA0320_AAG03709.1

cadC_AAB59153.1

czcP_ABF12829.1

cadB_1_AY048756

zupT/ygiE_AAA69208.1

dsbA_AAC43535.1

irlS_AFI69387.1

czcS_CAA67087.1

cznC_CBG40020.1

nccH_AAA65103.1

czcR_ABF12837.1

ychH_BAA36063.1

nccY_AAA65101.1

dsbA_AAC43520.1

czcC_CAA67082.1

dsbB_BAA36032.2

actR_ABR62097.1

zntA/yhhO_BAE77824.1

dsbA_AAC43532.1

czrB_CAB56470.1

mreA_AAN68577.1
