# Supplementary material for: Gut heavy metal and antibiotic resistome of humans living in the high Arctic
Source: Front Microbiol. 2024 Oct 30;15:1493803. doi: 10.3389/fmicb.2024.1493803 (PMC11557323; doi:10.3389/fmicb.2024.1493803)
Supplement: Supplementary file 4 [file Table_1.DOCX]

| Pair | Adjusted R^2^ | Cor |
| --- | --- | --- |
| Hg-Cd | 0.365 | 0.6138166 |
| Hg-Pb | 0.1285 | 0.380295 |
| Pb-Cd | -0.01362 | 0.07178035 |

Table S1. Linear regression analysis of correlation between the fecal concentrations of heavy metals.
