# Supplementary material for: Gut heavy metal and antibiotic resistome of humans living in the high Arctic
Source: Front Microbiol. 2024 Oct 30;15:1493803. doi: 10.3389/fmicb.2024.1493803 (PMC11557323; doi:10.3389/fmicb.2024.1493803)
Supplement: Supplementary file 5 [file Table_2.DOCX]

| **Heavy metal** | **Gene Name** | **log2FC** | **lfcSE** | **p-value** | **padj** |
| --- | --- | --- | --- | --- | --- |
| **Pb** | **lactose ABC transporter permease** | 7.54 | 1.36 | 1.37E-06 | 7.80E-03 |
| **Pb** | **hemagglutinin family protein** | -2.33 | 1.12 | 1.67E-06 | 7.80E-03 |
| **Pb** | **MULTISPECIES: DUF3987 domain-containing protein** | -2.43 | 1.14 | 2.37E-06 | 7.80E-03 |
| **Pb** | **electron transporter HydN** | -0.79 | 1.10 | 3.99E-06 | 9.83E-03 |
| **Pb** | **double-strand break repair protein AddB** | -3.66 | 1.23 | 9.41E-06 | 1.86E-02 |
| **Hg** | **2-hydroxy-acid oxidase** | 7.83 | 1.65 | 3.28E-06 | 1.74E-02 |
| **Hg** | **CHAD domain-containing protein** | 9.95 | 1.64 | 3.52E-06 | 1.74E-02 |
| **Hg** | **MULTISPECIES: gluconate transporter** | -6.92 | 1.69 | 6.67E-06 | 2.19E-02 |
| **Hg** | **MULTISPECIES: N-carbamoylputrescine amidase** | 7.59 | 1.65 | 1.11E-05 | 2.74E-02 |
| **Hg** | **MULTISPECIES: beta-hydroxyacyl-ACP dehydratase** | -5.70 | 1.68 | 1.69E-05 | 3.08E-02 |
| **Hg** | **Hydrogenase-4 component B / Formate hydrogenlyase subunit 3** | 8.87 | 1.56 | 1.88E-05 | 3.08E-02 |
| **Hg** | **MULTISPECIES: beta-glucanase** | 6.77 | 1.68 | 2.91E-05 | 4.09E-02 |
| **Hg** | **multidrug ABC transporter** | -4.22 | 1.69 | 3.64E-05 | 4.49E-02 |

*Table S2. Benjamin Hochberg significant association results of gut heavy metal concentration associated with gut microbiome RNA expression. All tests were carried out correcting for the first 3 PEER factos, individual, and whether the sample is from a winter month or not. No gene expression was significantly associated with cadmium. P-value QQ-plots showed in general well-controlled test statistics for lead and cadmium, a slight inflation for mercury).*
